# P-1956. COVID-19 in pediatric population in Peru, 2020-2023: national data analysis

**DOI:** 10.1093/ofid/ofae631.2115

**Published:** 2025-01-29

**Authors:** Brian Peña, Gabriel Carrasco, Theresa Ochoa, Rafaella Navarro

**Affiliations:** UPCH, Lima, Lima, Peru; UPCH, Lima, Lima, Peru; Instituto de Medicina Tropical Alexander von Humboldt, Universidad Peruana Cayetano Heredia, Lima, Lima, Peru; UPCH, Lima, Lima, Peru

## Abstract

**Background:**

Peru is the eighth country in South America with most COVID-19 cases (134 684 cases per million population) and the first country in the world with most COVID-19 deaths (6 595 deaths per million population). However, there is no systematic analysis of the prevalence and severity of COVID-19 in the pediatric and adolescent population in the country. This study aims to describe the distribution and disparities of COVID-19 in Peru.

**Figure:**
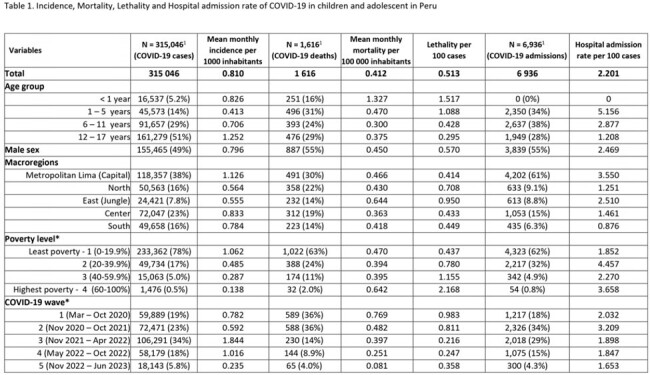

**Methods:**

We analyzed all COVID-19 cases, deaths, admissions, and vaccine doses administered reported in the open database of the Ministry of Health of Peru from March 2020 to June 2023. The main variables were the monthly number of COVID-19 cases, deaths, and hospital admissions. We calculated monthly incidence per 1,000 inhabitants, monthly mortality per 100,000 inhabitants, global lethality per 100 cases, and global hospitalization rate per 100 cases by sex, age group (under 1y, 1-5y, 6-11y, and 12-17y), poverty level (1 to 4 from least to highest poverty level), macroregion (metropolitan Lima, north, east [jungle], center, and south), and COVID-19 waves (1: Mar–Oct2020, 2: Nov2020–Oct2021, 3: Nov2021–Apr2022, 4: May–Oct2022, and 5: Nov2022–Jun2023).

**Figure:**
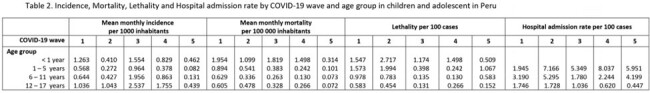

**Results:**

We analyzed 315,046 COVID-19 cases, 1,616 COVID-19 deaths, 6,936 hospital admissions and 16,476,930 vaccine doses administered. The mean monthly incidence were higher in adolescents (1.252), patients from the metropolitan Lima macroregion (1.126), from the lowest poverty level (1.062), and during the third and fourth waves (1.844 and 1.016). However, the mean monthly mortality were higher in children under 1 year (1.327), patients from the jungle macroregion (0.664), with the highest poverty level (0.642), and during the first and second COVID-19 waves (0.769 and 0.482). Lethality and hospital admission rates had similar distribution to monthly mortality (Tab 1). Analyzing COVID-19 cases by age group during each wave, we found that there are higher monthly incidence rates among adolescents and higher monthly mortality and global lethality rates in children under 1 year. (Tab 2, Fig 1 & 2)

**Figure 1.**
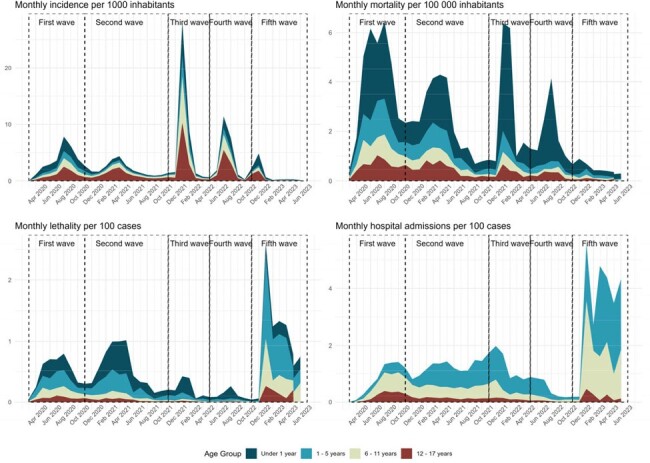
Monthly COVID-19 incedence, mortality, lethality and hospital admission rate from march 2020 to june 2023 in pediatric population in Peru

**Conclusion:**

COVID-19 in the pediatric population in Peru had great disparities, with the highest mortality among young infants, the poorest and children from the jungle.

**Figure 2.**
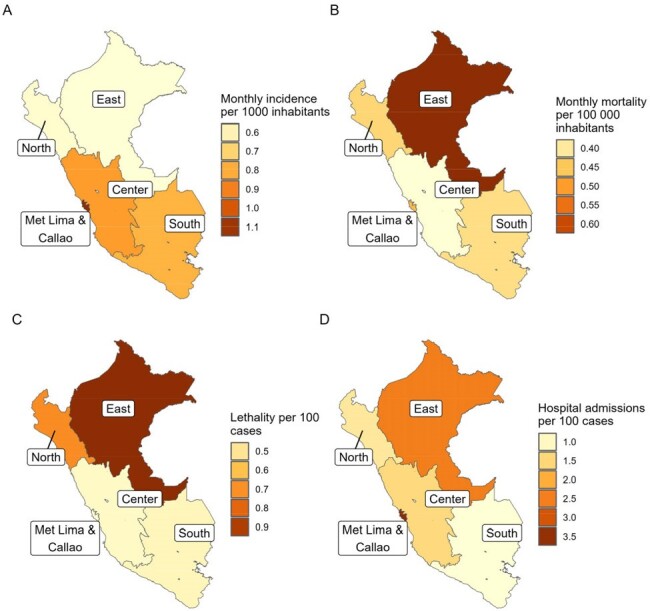
Monthly COVID-19 incidence, mortality, lethality and hospital admission rate by macroregion in children and adolescents from March 2020 to June 2023 in pediatric population in Peru. A: Mean monthly incidence per 1,000 inhabitants by macroregion B: Mean monthly mortality per 100,000 inhabitants by macroregion C: Lethality per 100 inhabitants by macroregion D: Hospital admission rate per 100 inhabitants by macroregion

**Disclosures:**

Brian Peña, n/a, Pfizer: Grant/Research Support Gabriel Carrasco, n/a, Pfizer: Grant/Research Support Theresa Ochoa, PhD, Pfizer: Grant/Research Support Rafaella Navarro, n/a, Pfizer: Grant/Research Support

